# Starvation-Induced Disruption of Nocturnal activity in the tropical house cricket, *Gryllodes sigillatus*

**DOI:** 10.17912/micropub.biology.001701

**Published:** 2025-09-17

**Authors:** Hugo Moebel, Eshani Yeragi, Isaac Jones, Alex Keene

**Affiliations:** 1 Biology, Texas A&M University; 2 Biology, Prairie View A&M University, Prairie View, Texas, United States

## Abstract

Feeding state potently modulates foraging behavior and locomotor activity. Here, we examined activity patterns across developmental stages in the nocturnal tropical house cricket or banded cricket, *Gryllodes sigillatus* . Adult males and females exhibited robust nocturnal behavior, with nocturnality emerging earlier in females during development. Starvation significantly reduced overall activity and abolished nocturnal activity patterns in females, but not in males. These results reveal sex-dependent differences in the developmental and feeding-state regulation of nocturnal behavior in *G. sigillatus* and lay the groundwork for future studies on how nutrient stress modulates behavior in this species.

**Figure 1. Ontogeny and sex differences in nocturnal activity of Gryllodes sigillatus and effects of Acute Food Deprivation:**
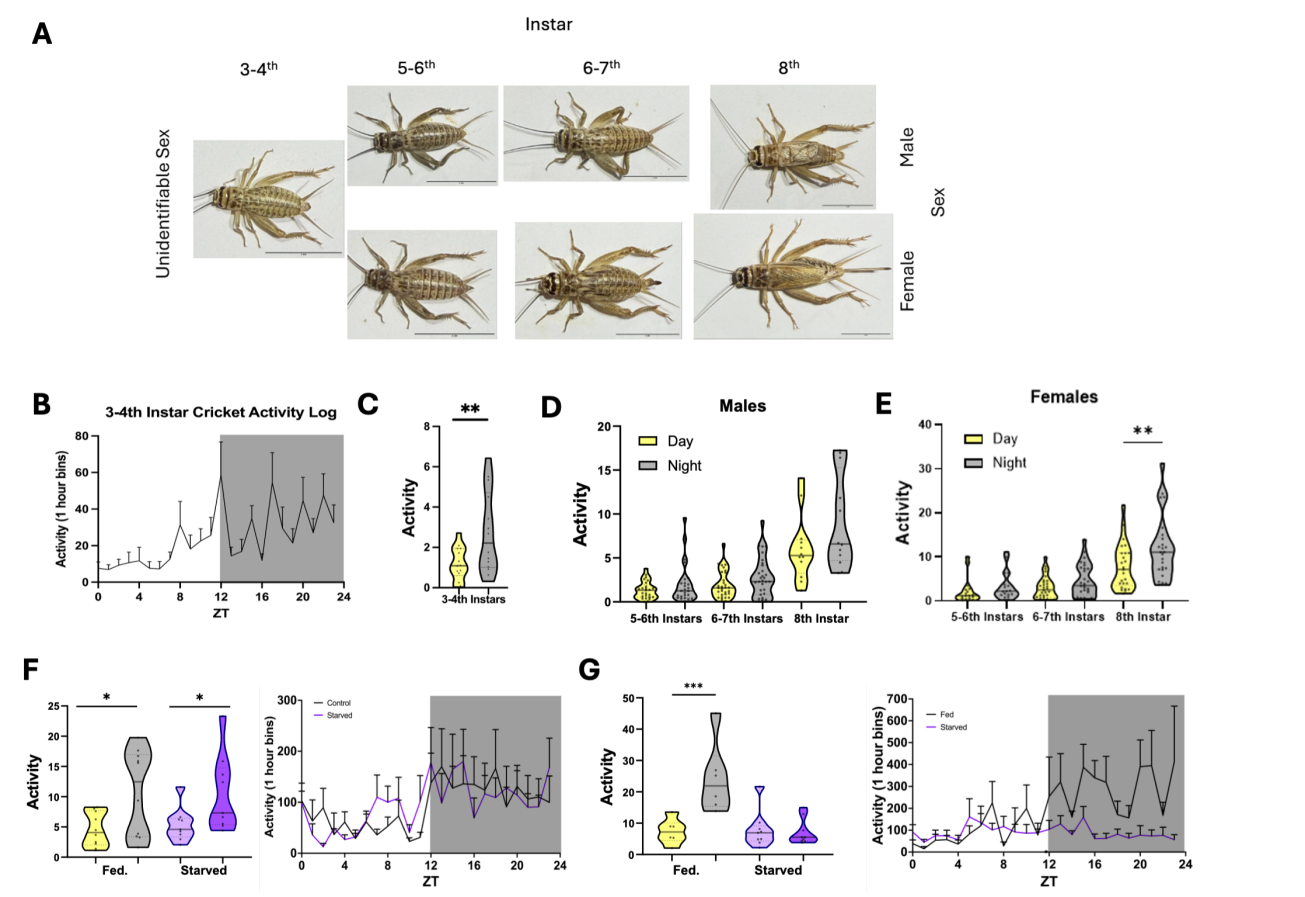
**(A)** Representative images of crickets from the 3–4th, 5–6th, 6–7th, and 8th+ instars, separated by sex when distinguishable. (B) Day vs. night activity of 3–4th instar crickets (n = 14) showed significantly higher activity during the night (p = 0.0018, paired t-test, t(13) = 3.905). **(C)** Average activity profile (mean ± SEM) of 3–4th instars over 24 h under 12:12 light: dark **(D)** Two-way ANOVA revealed a significant main effect of time-of-day F(5,100)=17.42,𝑝<0.0001, but no significant effect of instar stage F(30,100)=1.172, p=0.2755. Tukey’s post-hoc tests showed significantly increased activity at the 8th instar compared to earlier instars (p < 0.0001). However, day-night differences within each instar, including the 8th, were not significant after multiple testing correction (adjusted p = 0.0519) indicating a trend toward but not definitive emergence of nocturnality in late-stage males. **(E)** Significant main effects were observed for both instar stage F(28,110)=2.213, p=0.0019, and time-of-day 𝐹(5,110)=23.84, p<0.0001. Post-hoc Tukey’s tests revealed a significant Day vs. Night difference at the 8th instar (p = 0.0045), but not at earlier stages (p > 0.6) indicating a developmental shift in circadian behavior in female cricket. **(F)** Violin plots depict locomotor activity under fed (yellow = day, grey = night) and starved (light purple = day, dark purple = night) conditions in male 8th+ instar. In both fed (n = 10) and starved (n = 9) groups, paired t-test revealed significantly higher nighttime activity compared to daytime under fed conditions (t = 2.619, df = 9, p = 0.0278, n = 10, η² = 0.43), as well as under starved conditions (t = 2.902, df = 8, p = 0.0198, n = 9, η² = 0.51). Two-way ANOVA detected a significant main effect of condition (F (3,25) = 3.529, p = 0.0293), but no effect of group or interaction. Tukey’s post hoc tests did not yield significant pairwise differences after multiple comparison correction. **(G)** Violin plots showing locomotor activity in female 8th+ instar cricket across fed and starved conditions during day and night. Starved female crickets (purple) slept less than fed counterparts during the night (grey), and nocturnal activity preference was abolished (n = 9 for starved, n = 6 for fed). A paired t-test showed significantly increased nighttime activity compared to daytime under fed conditions (t = 5.255, df = 5, p = 0.0033, n = 6, η² = 0.85), while no significant day-night difference was observed under starvation (t = 0.410, df = 8, p = 0.693, n = 9).Two-way ANOVA revealed a significant main effect of condition (p = 0.0293), with Tukey’s post hoc tests confirming that Fed Night females were significantly more active than Fed Day females (p = 0.0005), Starved Day females (p = 0.0010), and Starved Night females (p = 0.0007). No significant differences were found among the remaining groups after correction. Asterisks indicate significance levels: p < 0.05 (*), p < 0.01 (**), p < 0.001 (***); ns = not significant.

## Description

Crickets are widely studied for their complex behaviors that are modulated by social interactions, developmental state, and life history (Huber et al., 1998). The tropical house cricket, *Gryllodes sigillatus* , is an emerging model due to its impact as an agricultural pest, use as pet food, and its sophisticated behaviors (Hall et al., 2017; Kong et al., 2024). Previous studies have shown that adult male *G. sigillatus* exhibit robust nocturnal locomotor rhythms that persist under constant darkness (Abe et al., 1997). Here, we examined the establishment of nocturnal activity patterns across development, and how these activity patterns are modulated by feeding state.

Crickets are highly sexually dimorphic in behavior and morphology (Huber et al., 1998). To examine whether activity patterns differed across sexes, we measured locomotor patterns in 12:12 light:dark cycles in males and females across development (Fig 1A). At the 3 ^rd^ /4 ^th^ instar stage when morphological sexing is challenging, activity was robustly nocturnal (Fig 1B,C). We then measured activity in males and females at three distinct developmental time points. Analysis of day-night differences in activity using a repeated measures ANOVA did not reveal significant differences at any timepoint in males, though there was a trend towards nocturnal activity that was present in 8 ^th^ instars (Fig 1D. In female crickets, significantly greater nighttime activity was detected in 8 ^th^ instar females, with trends towards increased nighttime activity present at 6 ^th^ /7 ^th^ instar (Fig 1E). Together, these findings suggest nocturnal behavior is more robust in female crickets than in males.

In diurnal *Drosophila* , food-deprivation or metabolic stress induces hyperactivity, but this has not been investigated in a nocturnal insect (Keene et al., 2010; Lee & Park, 2004). To investigate the effects of food deprivation in *G. sigillatus* we starved 8 ^th^ instar male and female crickets for 24-hours. Starved males retained nocturnal activity patterns, which was significant with a paired student t-test ( Fig. 1F ). Conversely, starvation suppressed nighttime sleep in females and abolished nocturnal activity patterns (Fig 1G). These findings suggest that females respond to starvation by suppressing activity and are more sensitive to nutrient-deprivation than males.

Locomotor activity has been investigated in multiple cricket species (Tomioka, 2014). In *G. sigillatus* , activity is robustly nocturnal and persists under conditions of constant darkness (Abe et al., 1997). In addition to locomotion, crickets exhibit circadian rhythms in stridulation that are disrupted by exposure to artificial light (Levy et al., 2024). The sensitivity to pesticides is also under circadian regulation, indicating widespread effects of light-dark cycles on physiological processes (Levy et al., 2024; Piechowicz & Przemysław, 2014). The observation that starvation disrupts locomotor activity in females but not males may reflect physiological differences in metabolic response or underlying genetic or neural mechanisms that regulate behavior in a sex-specific manner. Future studies investigating the relationship between food availability, foraging behavior, and activity patterns in *G. sigillatus* and other species could provide insight into the mechanisms underlying sex-specific responses to nutrient stress.

Here, we identify robust sex-specific differences in activity in *G. sigillatus* , with females exhibiting more pronounced nocturnal activity patterns under fed conditions. These differences may reflect underlying physiological trade-offs. Prior studies in crickets have demonstrated that nutrient availability strongly influences physiology, development, and behavior, indicating that sex-specific activity patterns could emerge because of differential energetic allocation or reproductive investment (Clark et al., 2014) *.* These differences extend to the acute behavioral effects of starvation. For example, fasted Texas field crickets ( *Gryllus texensis* ) are more likely to choose shelter during oviposition assays than fed counterparts, suggesting that nutrient limitation alters reproductive decision-making and risk sensitivity (Stahlschmidt et al., 2014). Therefore, it is possible that the reduced nighttime activity that we observe is reflective a diminished foraging behavior to lower predation risk.

In many insects, sleep and activity patterns are regulated by immune function and it is possible that the effects of diet on activity are mediated by sex-specific changes in immune function. Female *G. texensis* fed a high-quality diet have larger body size, yet poorer disease resistance than counterparts fed a low-quality diet, leading to the hypothesis that female crickets prioritize immune function under nutrient-poor conditions (Kelly & Tawes, 2013) *.* Therefore, sex-specific differences in immune response may contribute to the loss of nighttime activity in starved *G. sigillatus* females.

Finally, in many specifies ranging from *Drosophila* through humans, starvation results in reduced sleep and increased activity. While these changes have been attributed to a sleep-foraging tradeoff, starvation sensitivity and foraging vary dramatically both between *Drosophila* species, and within outbred populations of *D. melanogaster* (Brown et al., 2018). Therefore, it is possible that unique reproductive demands on female *G. sigillatus* underlie suppression locomotor activity under starved conditions. Recently, machine learning approaches have been applied to enable simultaneous and automated quantification of multiple behaviors in crickets, including sleep and feeding (Hayakawa et al., 2024). Applying these analyses to study the roles of sex and developmental stage in circadian regulation of activity could reveal novel interactions between feeding and the control of locomotor behavior.

## Methods

**Husbandry** : Crickets were obtained through Carolina Biological Supply and maintained in an insulated room at 29 ± 1 °C and 50–60% relative humidity using an automatic humidifier. A 12:12 light-dark cycle was implemented from 10:00 AM to 10:00 PM, with LED lighting controlled by a switch timer. Crickets were housed in 10-gallon tanks with a dirt substrate and provided with egg cartons for shelter and access to resources. All life stages and sexes were housed together. Crickets were fed Mazuri Cricket Diet in powdered form every 2–3 days and placed in a food dish (Exo Terra Cricket Pen) separate from the water source. Hydration was provided via ABDragons Insect Water Crystals (1 teaspoon per 8 oz of water), placed in Glad containers, and refreshed every 2–3 days along with the food. Crickets were staged and sexed as previously described in Kong et al., 2024.

**Data Collection** : Experiments were conducted in an incubator (Powers Scientific) set to 29 ± 1 °C with 50–60% humidity. Crickets were given 14–16 hours of acclimation in activity monitors prior to data collection. Crickets were singly housed in 25 mm Locomotor Activity Monitor (LAM) tubes (TriKinetics Inc., MA) to prevent social interaction and ensure individual activity tracking. Each tube contained a water crystal coated with Mazuri Cricket Diet powder. Following a 14–16 h acclimation period, locomotor activity was recorded using infrared beam breaks at 5-minute intervals under a 12:12 light–dark cycle at 29 ± 1 °C and 50–60% humidity.

**Statistics:** All statistical analyses were performed using GraphPad Prism (v10.0). For early developmental stages (3–4th instars), day vs. night activity was compared using two-tailed paired Student’s t-tests ( Figure 1B &C). For analyses across instar stages, activity data were analyzed separately for males and females using two-way ANOVAs. In these analyses, time of day (day vs. night) was treated as a within-subjects factor (as the same individuals were recorded during both periods), and instar stage served as a between-subjects factor. Significant main effects and interactions were followed by Tukey’s multiple comparisons post hoc tests to determine p-values for specific pairwise comparisons. For the starvation experiments (Figures F & G), activity of 8th+ instar male and female crickets was analyzed using two-tailed paired t-tests to compare day vs. night activity within each feeding condition (fed or starved), treating time of day as a within-subjects factor. Additionally, two-way ANOVAs were used to assess the main effects of feeding state (fed vs. starved) and time of day, with Tukey’s post hoc tests for multiple comparisons across the four groups (Fed Day, Fed Night, Starved Day, Starved Night).
